# Proposed prognostic subgroups and facilitated clinical decision-making for additional locoregional radiotherapy in de novo metastatic nasopharyngeal carcinoma: a retrospective study based on recursive partitioning analysis

**DOI:** 10.1186/s13014-022-02168-2

**Published:** 2023-01-21

**Authors:** Yuyi Yao, Xuesong Sun, Huageng Huang, Zhao Wang, Xiaojie Fang, Meiting Chen, Zegeng Chen, Huawei Weng, Chengcheng Guo, Huangming Hong, He Huang, Tongyu Lin

**Affiliations:** 1 Department of Medical Oncology, State Key Laboratory of Oncology in South China, Guangdong Key Laboratory of Nasopharyngeal Carcinoma Diagnosis and Therapy, Sun Yat-sen University Cancer Center, 651 Dongfeng East Road, Guangzhou, 510060 P. R. China; 2 Department of Nasopharyngeal Carcinoma, State Key Laboratory of Oncology in South China, Guangdong Key Laboratory of Nasopharyngeal Carcinoma Diagnosis and Therapy, Sun Yat-sen University Cancer Center, 651 Dongfeng East Road, Guangzhou, 510060 P. R. China; 3 Senior Ward and Phase I Clinical Trial Ward, Sichuan Cancer Hospital & Institute, Sichuan Cancer Center, School of Medicine, University of Electronic Science and Technology of China, No.55, Section 4, South Renmin Road, Chengdu, People’s Republic of China

**Keywords:** De novo metastatic nasopharyngeal carcinoma, Recursive partitioning analysis, Risk stratification, Locoregional radiotherapy, Overall survival

## Abstract

**Background:**

The high heterogeneity of de novo metastatic nasopharyngeal carcinoma (dmNPC) makes its prognosis and treatment challenging. We aimed to accurately stage dmNPC and assess the patterns of treatment strategies for different risk groups.

**Methods:**

The study enrolled a total of 562 patients, 264 from 2007 to 2013 in the training cohort and 298 from 2014 to 2017 in the validation cohort. Univariate and multivariate Cox regression analyses were conducted to determine the independent variables for overall survival (OS). Recursive partitioning analysis (RPA) was applied to establish a novel risk-stratifying model based on these variables.

**Results:**

After pairwise comparisons of OS, three risk groups were generated: low-risk (involved lesions ≤ 4 without liver involvement), intermediate-risk (involved lesions ≤ 4 with liver involvement or involved lesions > 4 with Epstein–Barr virus (EBV)-DNA < 62,000 copies/ml), and high-risk (involved lesions > 4 with EBV-DNA > 62,000 copies/ml). The 3-year OS rate differed significantly between groups (80.4%, 42.0%, and 20.4%, respectively, all *P* < 0.05). Adding locoregional intensity-modulated radiotherapy (LRRT) followed by palliative chemotherapy (PCT) resulted in a significant OS benefit over PCT alone for the low- and intermediate-risk groups (*P* = 0.0032 and *P* = 0.0014, respectively). However, it provided no survival benefits for the high-risk group (*P* = 0.6). Patients did not benefit from concurrent chemotherapy during LRRT among the three subgroups (*P* = 0.12, *P* = 0.13, and *P* = 0.3, respectively). These results were confirmed with the validation cohort.

**Conclusions:**

The novel RPA model revealed superior survival performance in subgroup stratification and could facilitate more effective treatment strategies for dmNPC.

**Supplementary Information:**

The online version contains supplementary material available at 10.1186/s13014-022-02168-2.

## Background

Nasopharyngeal carcinoma (NPC) is a highly aggressive and metastatic malignancy that is spread throughout in southern China and Southeast Asia [1]. As reported in Global Cancer Statistics 2020, more than 130,000 patients are diagnosed worldwide, and over 80,000 new deaths occur annually [2]. Due to the insidious site of NPC and a lack of symptoms at the initial stage of the disease, it is typically not diagnosed until the disease has progressed to an advanced state. Almost 10% of patients already have distant metastases when diagnosed, and the overall survival (OS) is approximately 15 months when only receive palliative chemotherapy (PCT) is performed [1].

De novo metastatic NPC (dmNPC) is a heterogeneous population in which the tumor-node-metastasis (TNM) staging system is incapable of risk stratification. More expedient risk stratification is needed to identify individuals before treatment initiation. In addition, NPC is one of the epithelial cell malignancies most tightly associated with Epstein–Barr virus (EBV). It has been noted that the quantification of plasma EBV-DNA is strongly correlated with the stage of NPC and is a reliable prognostic marker [3, 4]. Combining plasma EBV-DNA concentration with clinical-anatomical factors may help better stratify individuals with distant metastasis into different disease subtypes and predict individualized clinical prognoses.

Furthermore, there is no consensus on the optimal regimen for dmNPC. Recently, the regimen of PCT followed by locoregional intensity-modulated radiotherapy (LRRT) has been shown to incredibly prolong the OS over PCT alone after PCT [5]. However, the heterogeneity of the anatomically different metastatic lesions between patients is often closely related to their prognosis; selection of the most appropriate patients who would benefit from additional LRRT is an urgent problem. In addition, no consensus has yet to be reached regarding LRRT combined with concurrent chemotherapy (CCT) after PCT in dmNPC either, and the survival benefit is indeterminate.

Recursive partitioning analysis (RPA) is a practical cancer staging tool for predicting patient survival guiding optimal medical decision-making via combinations of clinical variables [6]. Here, we established an RPA model for individualized prognostic prediction in dmNPC and assessed whether additional LRRT could be more beneficial in terms of survival than PCT alone in each risk group.

## Methods

### Study participants

**Fig. 1:**
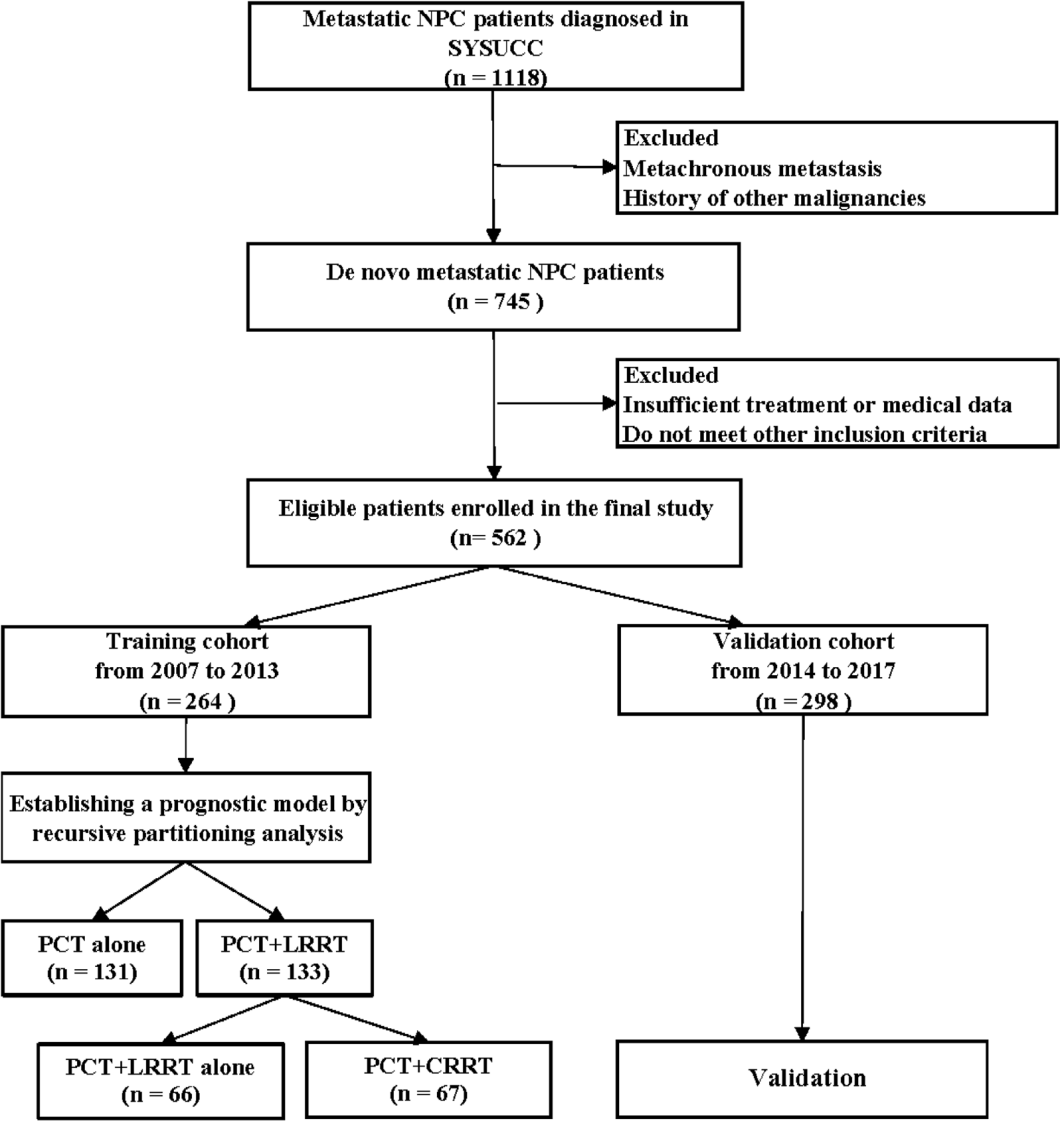
Development and validation of a prognostic model for de novo metastatic nasopharyngeal carcinoma (dmNPC) patients

From January 2007 to December 2017, 562 patients with distant metastatic disease at diagnosis received treatment at the Sun Yat-Sen University Cancer Center (SYSUCC). 264 were enrolled from 2007 to 2013 for the training cohort, while 298 patients were enrolled from 2014 to 2017 for the validation cohort. The inclusion criteria were presented as follows: (1) newly diagnosed biopsy-proven NPC; (2) distant metastasis based on histological or radiographic evidence; (3) measurable disease evaluated defined by the Response Evaluation Criteria in Solid Tumors (RECIST) 1.1; (4) Eastern Cooperative Oncology Group (ECOG) performance status less than or equal to 1 point; (5) pretreated hematological results including plasma EBV-DNA copy numbers; (6) adequate liver and kidney functions throughout the study; (7) platinum-based PCT regimen used as recommended by the National Comprehensive Cancer Network guidelines with or without LRRT; and (8) complete follow-up data. The exclusion criteria were presented as follows: (1) a history of prior or concurrent malignancies; (2) serious concurrent disease that would compromise safety; (3) congenital or acquired immunodeficiency; (4) previous systemic therapy for NPC; and (5) pregnancy or lactation. All patients were evaluated prior to treatment, including physical examination, nasopharyngoscopy, contrast-enhanced magnetic resonance imaging (MRI) of the head and neck, abdominal ultrasonography, chest and abdominal computed tomography (CT) scans, and emission computed tomography (ECT) bone scans. Positron emission tomography (PET)-CT was performed when necessary. The flowchart of patient enrollment is provided in Fig. 1. Patients in these two cohorts were restaged according to the American Joint Committee on Cancer (AJCC) 8th edition.

### Treatment regimen

Patients received platinum-based PCT with a regimen consisting of docetaxel (75 mg/m^2^ intravenously [IV] on day 1) plus cisplatin (20–25 mg/m^2^ IV on days 1–3), or docetaxel (60–75 mg/m^2^, IV on day 1) plus cisplatin (20–25 mg/m^2^, IV on days 1–3) plus 5-fluorouracil (500–800 mg/m^2^, continuous IV infusion for 24 h on days 1–5), or cisplatin (20–30 mg/m^2^ IV on days 1–3) plus 5-fluorouracil (800–1000 mg/m^2^ continuous IV infusion for 24 h on days 1–5), or gemcitabine (800–1000 mg/m^2^ IV on days 1, 8) plus cisplatin (20–30 mg/m^2^ IV on days 1–3), or others. Patients were enrolled if they received at least two cycles of PCT and completed follow-up. The radiotherapy regimen was intensity-modulated radiotherapy (IMRT), the details of which have been published previously [7, 8]. The gross target volume of the nasopharynx (GTVnx) and neck lymph nodes involvement (GTVnd) were determined based on MRI and physical examinations. The high-risk clinical target volume (CTV1) was defined as the volume of GTVnx plus a 5–10 mm margin, whereas the preventive clinical target volume (CTV2) was defined as the volume of CTV1 plus a 5–10 mm margin. The prescribed radiotherapy doses were 66–72 Gy to the primary tumor, 60–70 Gy to the lymph node–positive area and 50–60 Gy to the lymph node–negative area. An approximately a 10% dose reduction was allowed. The prescribed dose of RT was given in 1.8–2.3 Gy per day for 5 days per week. The main platinum-based regimen of CCT during LRRT included cisplatin 80–100 mg/m^2^ IV every 3 weeks or 30–40 mg/m^2^ IV weekly, nedaplatin 80 mg/m^2^ IV every 3 weeks, carboplatin (area under the free carboplatin plasma concentration versus time curve, AUC 6) every 3 weeks or (AUC 2) IV weekly. The regimen was chosen at the discretion of the physicians. The treatment scheme is shown in Table 1.

**Table 1:** Clinical characteristics

	Training cohort No. (%)	Validation cohort No. (%)	*P*
Total	264	298	
Age (years)			
< 47	130 (49.2)	148 (49.7)	0.920
≥ 47	134 (50.8)	150 (50.3)	
Sex			
Male	218 (82.6)	252 (84.6)	0.525
Female	46 (17.4)	46 (15.4)	
Family history of NPC			
No	245 (92.8)	277 (93.0)	0.945
Yes	19 (7.2)	21 (7.0)	
Smoking history			
No	157 (59.5)	191 (64.1)	0.260
Yes	107 (40.5)	107 (35.9)	
Drinking history			
No	239 (90.5)	278 (93.3)	0.229
Yes	25 (9.5)	20 (6.7)	
Tumor stage*			
T1-T2	42 (15.9)	31 (10.4)	0.053
T3-T4	222 (84.1)	267 (89.6)	
Node stage*			
N0-N1	49 (18.6)	42 (14.1)	0.151
N2-N3	215 (81.4)	256 (85.9)	
Bone involvement			
No	83 (31.4)	82 (27.5)	0.308
Yes	181 (68.6)	216 (72.5)	
Lung involvement			
No	200 (75.8)	224 (75.2)	0.871
Yes	64 (24.2)	74 (24.8)	
Liver involvement			
No	185 (70.1)	223 (74.8)	0.207
Yes	79 (29.9)	75 (25.2)	
Distant lymph node involvement			
No	220 (83.3)	230 (77.2)	0.068
Yes	44 (16.7)	68 (22.8)	
Number of involved organs			
Single	192 (72.7)	199 (66.8)	0.126
Multiple	72 (27.3)	99 (33.2)	
Number of involved lesions			
Single	67 (25.4)	63 (21.1)	0.234
Multiple	197 (74.6)	235 (78.9)	
EBV-DNA status			
EBV-DNA negative	24 (9.1)	17 (5.7)	0.123
EBV-DNA positive	240 (90.9)	281 (94.3)	
Therapy regimens			
PCT	131 (49.6)	112 (37.6)	0.004
PCT plus IMRT	133 (50.4)	186 (62.4)	
Chemotherapy cycle			
< 4	27 (10.2)	19 (6.4)	0.096
≥ 4	237 (89.8)	279 (93.6)	
Radiotherapy dose of loco-regional			
< 66 Gy	2 (1.5)	1 (0.5)	0.573
≥ 66 Gy	131 (98.5)	185 (99.5)	
Pattern of Radiotherapy			
LRRT alone	66 (49.6)	79 (42.5)	0.206
CCT plus LRRT	67 (50.4)	107 (57.5)	

*NPC* nasopharyngeal carcinoma, *PCT* palliative chemotherapy, *IMRT* intensity-modulated radiotherapy, *LRRT* locoregional intensity-modulated radiotherapy, *CCT* concurrent chemotherapy, *EBV* Epstein–Barr virus, *No.* Number

^*^According to the 8th TNM staging system

### Outcome evaluation and follow-up

The primary endpoint was OS, which was calculated from the date of pathological diagnosis to death or date of the last contact. Follow-up visits consisted of fiberoptic nasopharyngoscopy, MRI of the head and neck, abdominal ultrasonography, CT scan of the chest/abdomen, bone scan, or PET/CT. The tumor response was evaluated independently by two experienced radiologists according to RECIST version 1.1 criteria [9]. The objective response rate (ORR) was defined as the percentage of patients with confirmed complete responses (CR) or partial responses (PR) to PCT. The evaluation of tumor response is performed every two cycles of PCT. Plasma EBV-DNA concentrations were measured prior to treatment via real-time quantitative polymerase chain reaction (PCR) analysis. After the treatment was completed, patients were assessed every 3 months for the first 3 years, then every 6 months for up to 5 years, and annually thereafter.

### Statistical analysis

Survival outcomes were estimated by the Kaplan–Meier method, and the log-rank test was used to assess survival differences between groups. Univariate and multivariate analyses for OS were conducted by the Cox proportional hazards model, and hazard ratios (HRs) with two-sided 95% confidence intervals (CIs) were calculated. The optimum cutoff values were calculated through the analysis of the receiver operating characteristic (ROC) curve analysis. Using the independent prognostic factors identified by the multivariate analysis, RPA was performed to develop predictive models for risk stratification. Construction of the prediction model by RPA analysis was performed by autoRPA, an algorithm located on a web server [10] (http://rpa.renlab.org). It can build a decision tree from survival data through the RPA algorithm and log-rank statistics, providing a reasonable basis for making appropriate clinical decision [11]. Time-dependent area under the curve (tAUC) was calculated to evaluate the discriminatory ability of the model, and decision curve analysis (DCA) was applied to evaluate the clinical net benefit of the model for predicting prognosis. We compared the associations between categorical variables using the chi-square test and Fisher’s exact test. All analyses were carried out using R version 4.0.2, and SPSS 25.0 software. All statistical tests were two-sided, and *P* < 0.05 was considered statistically significant.

## Result

### Patient characteristics

In our study, 562 patients were retrospectively recruited, with 264 patients in the training cohort and 298 in the validation cohort. The median age of the entire population was 47 years (interquartile range from 40 to 55 years). Overall, most patients were male in both cohorts (82.6% and 84.6%). A total of 240 patients (90.9%) in the training group and 282 (94.3%) in the validation group had detectable EBV-DNA in plasma before treatment. Most patients in both the training cohort (181 [68.6%]) and validation cohort (216 [72.5%]) had bone metastases. 237 patients (89.8%) and 279 (93.6%) received four received four or more cycles of PCT, respectively. The ORR to PCT was 65.2% (172 out of 264 patients) in the training cohort, and 64.0% (191 out of 298 patients) in the validation cohort, respectively. The median follow-up time was 94.9 months (interquartile range, 62.8 to 116.8 months) in the training cohort and 51.8 months (interquartile range, 41.2 to 66.4 months) in the validation cohort. The characteristics of the two cohorts are presented in Table 1.

### Univariate and multivariate analysis

We performed ROC curve analyses to determine the cutoff values of the continuous variables in the training cohort. Based on ROC curve analyses, we set the optimal age to 52 and divided the patients into old and young groups. The cutoff value of the number of pretreated plasma EBV-DNA copies was 62,000 copies/ml. Similarly, patients were stratified into two groups based on the number of involved lesions (≤ 4 and > 4). Univariable and multivariable Cox proportional hazard regression results for OS are listed in Table 2. Through univariate Cox regression analyses, age, EBV-DNA copy number, number of involved lesions, number of involved organs, distant lymph node involvement, tumor response to PCT, and liver involvement were significantly related to OS. Multivariate Cox proportional hazards regression analysis was performed with the variables considered significant in the univariate Cox regression analysis (P ≤ 0.05). Finally, pretreated plasma EBV-DNA copy number (copies/ml) (≥ 62,000 vs. < 62,000; HR 1.559, 95% CI: 1.104–2.202, *P* = 0.012), number of involved lesions (> 4 vs. ≤ 4; HR 2.302, 95% CI: 1.621–3.268, *P* < 0.001), liver involvement (yes vs. no; HR 2.055, 95% CI: 1.441–931, *P* < 0.001), tumor response to PCT (SD + PD vs. PR + CR; HR 1.558, 95% CI: 1.110–2.187, *P* = 0.010) and age (> 52 vs. ≤ 52; HR 1.018, 95% CI: 1.003–1.034, *P* = 0.018) remained independent prognostic factors. The number of involved organs (*P* = 0.710) and distant lymph node involvement (*P* = 0.585) were excluded.

**Table 2:** Univariable and multivariable analyses of the training group

Characteristics	Univariate analysis		Multivariate analysis	
	HR (95% CI)	*P*	HR (95% CI)	*P*
Age (years)	1.486 (1.075–2.056)	**0.017**	1.018 (1.003–1.034)	**0.018**
≤ 52				
> 52				
Gender	1.065 (0.705–1.607)	0.765		
Male				
Female				
Family history of NPC	0.979 (0.515–1.861)	0.949		
No				
Yes				
Smoking history	1.193 (0.863–1.647)	0.285		
No				
Yes				
Tumor stage	1.284 (0.823–2.002)	0.270		
T1-T2				
T3–T4				
Node stage	1.519 (0.986–2.341)	0.058		
N0-N1				
N2-N3				
EBV DNA copies/ml	2.252 (1.626–3.119)	** < 0.001**	1.559 (1.104–2.202)	**0.012**
< 62,000				
≥ 62,000				
No. of involved organs	2.705 (1.919–3.813)	** < 0.001**		
Single				
Multiple				
No. of involved lesions	3.227 (2.325–4.480)	** < 0.001**	2.302 (1.621–3.268)	** < 0.001**
≤ 4				
> 4				
Liver involvement	2.826 (2.023–3.948)	** < 0.001**	2.055 (1.441–2.931)	** < 0.001**
No				
Yes				
Bone involvement	1.245 (0.874–1.773)	0.225		
No				
Yes				
Lung involvement				
No	0.683 (0.455–1.024)	0.065		
Yes				
Distant lymph node involvement	1.733 (1.153–2.605)	**0.008**		
Yes				
No				
Tumor Response to PCT	2.115 (1.523–2.935)	< 0.001	1.558 (1.110–2.187)	**0.010**
PR/CR				
SD/PD				

*NPC* nasopharyngeal carcinoma, *PCT* palliative chemotherapy, *IMRT* intensity-modulated radiotherapy, *EBV* Epstein–Barr virus, *No.* Number, *CR* complete response, *PR* partial response, *PD* disease progression, *SD* stable disease

Bold values were considered statistically significant

### Risk stratification by recursive partitioning analysis

**Fig. 2:**
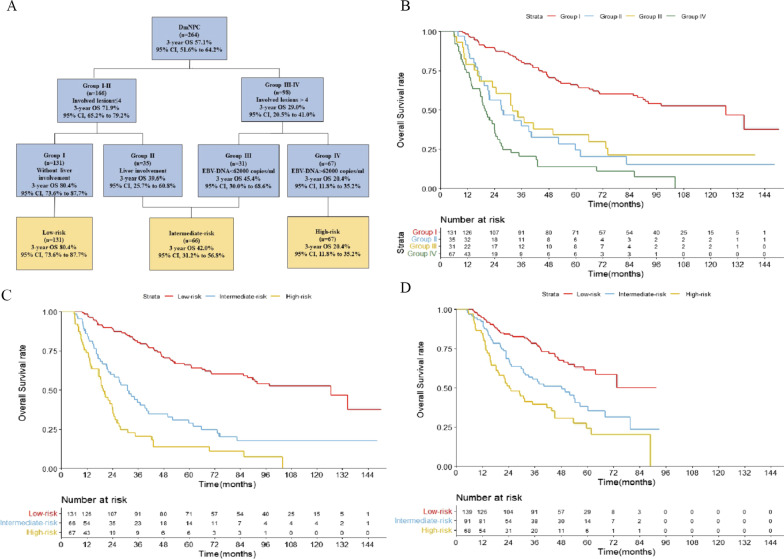
Staging procedure for de novo metastatic nasopharyngeal carcinoma (dmNPC) by recursive partitioning analysis in the training cohort (**A**); Kaplan–Meier survival curves for distinct risk factors in the training group (**B**); Kaplan–Meier survival curves based on the risk stratifications in the training group (**C**) and validation group (**D**)

The risk factors significant in multivariate Cox regression analysis (P ≤ 0.05) were subjected to the autoRPA stratification algorithms. A total of 264 patients were stratified through the autoRPA modeling system into one of four groups: Group I (involved lesions ≤ 4 without liver involvement); Group II (involved lesions ≤ 4 with liver involvement); Group III (involved lesions > 4 with EBV-DNA < 62,000 copies/ml); and Group IV (involved lesions > 4 with EBV-DNA > 62,000 copies/ml) (Fig. 2A). Next, a pairwise comparison did not reveal any significant difference in OS between the second and third groups (*P* = 0.606) (Fig. 2B). Therefore, we combined Group II and Group III into the same group (intermediate-risk group). Group I and group IV served as the low-risk and high-risk groups, respectively, and then all the groups were compared in pairs. A significant difference in survival was observed among the three groups; the 3-year OS rates of the low-, intermediate- and high-risk groups were 80.4%, 42.0% and 20.4%, respectively (all *P* < 0.05) (Fig. 2C). The validation cohort was then used to validate the stratification model. We classified the validation cohort into the 3 different risk groups based on the model, and the 3-year OS rate was significantly different between groups (78.2%, 55.3%, and 39.4%, respectively, *P* < 0.05) (Fig. 2D).

### Performance of the proposed RPA model in patient classification

**Fig. 3:**
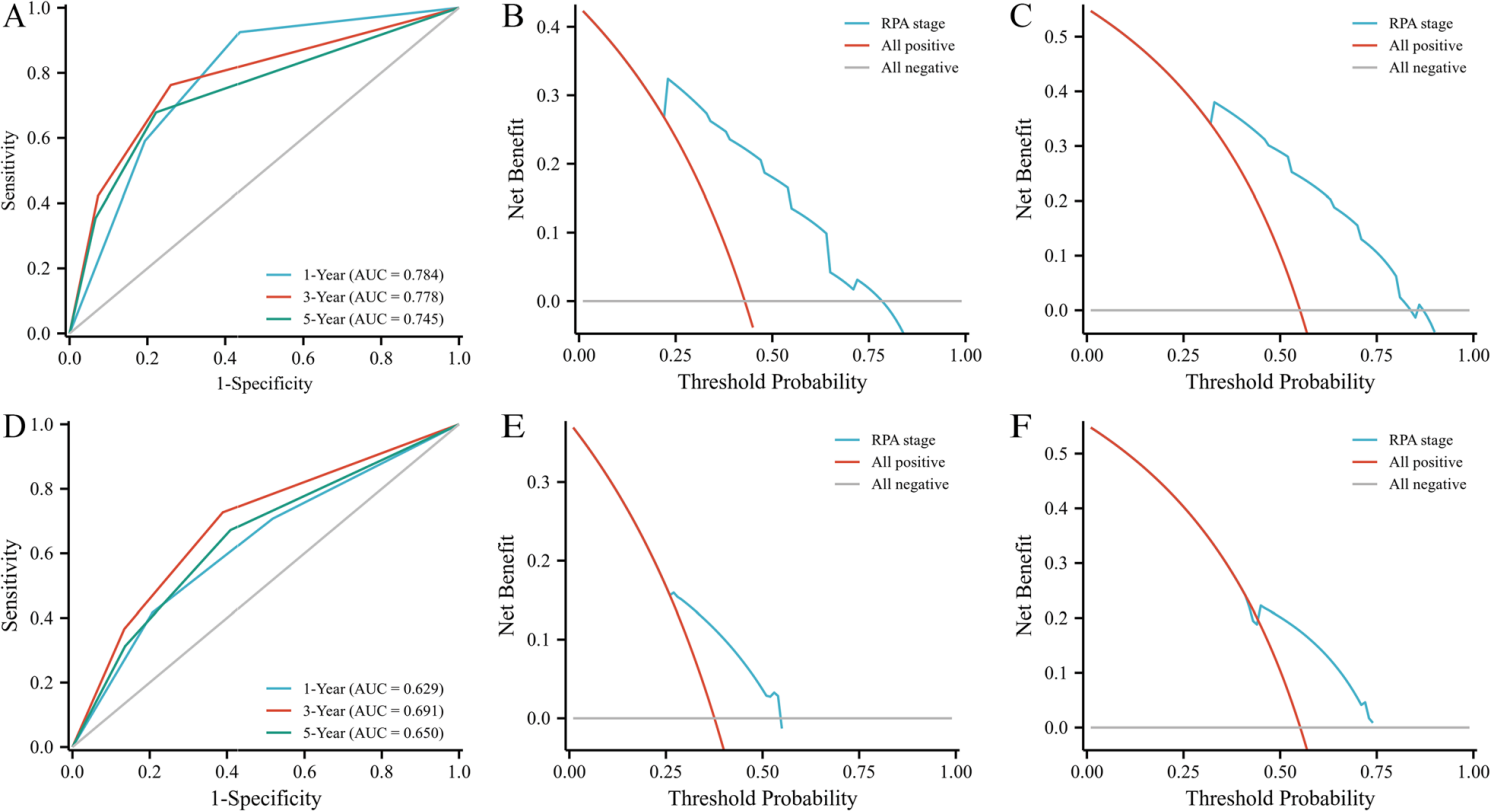
Performance of the Recursive partitioning analysis model according to the time-dependent area under the curve at 1-, 3-, and 5-year in the training cohort (**A**) and validation cohort (**D**). Decision curve analysis at 3 and 5 years in the training cohort (**B**, **C**) and validation cohort (**E**, **F**)

To evaluate the prognostic value of the novel RPA model for dmNPC, we constructed ROC curves and calculated the tAUC. In the training cohort, the 1-year, 3-year, and 5-year tAUCs of this RPA model was 0.784, 0.778, and 0.745, respectively (Fig. 3A). The the 1-year, 3-year, and 5-year tAUCs of the model was 0.629, 0.691, and 0.650 in the validation cohort (Fig. 3D). The 3- and 5-year DCA for the model in both the training and validation groups is visualized in Fig. 3B, C, E and F. The analysis demonstrated that the RPA staging system provided a more significant net clinical benefit than the ‘‘treat all” or ‘‘treat none” strategies in these cohorts.

### Efficacy of additional LRRT in variant risk groups

**Fig. 4:**
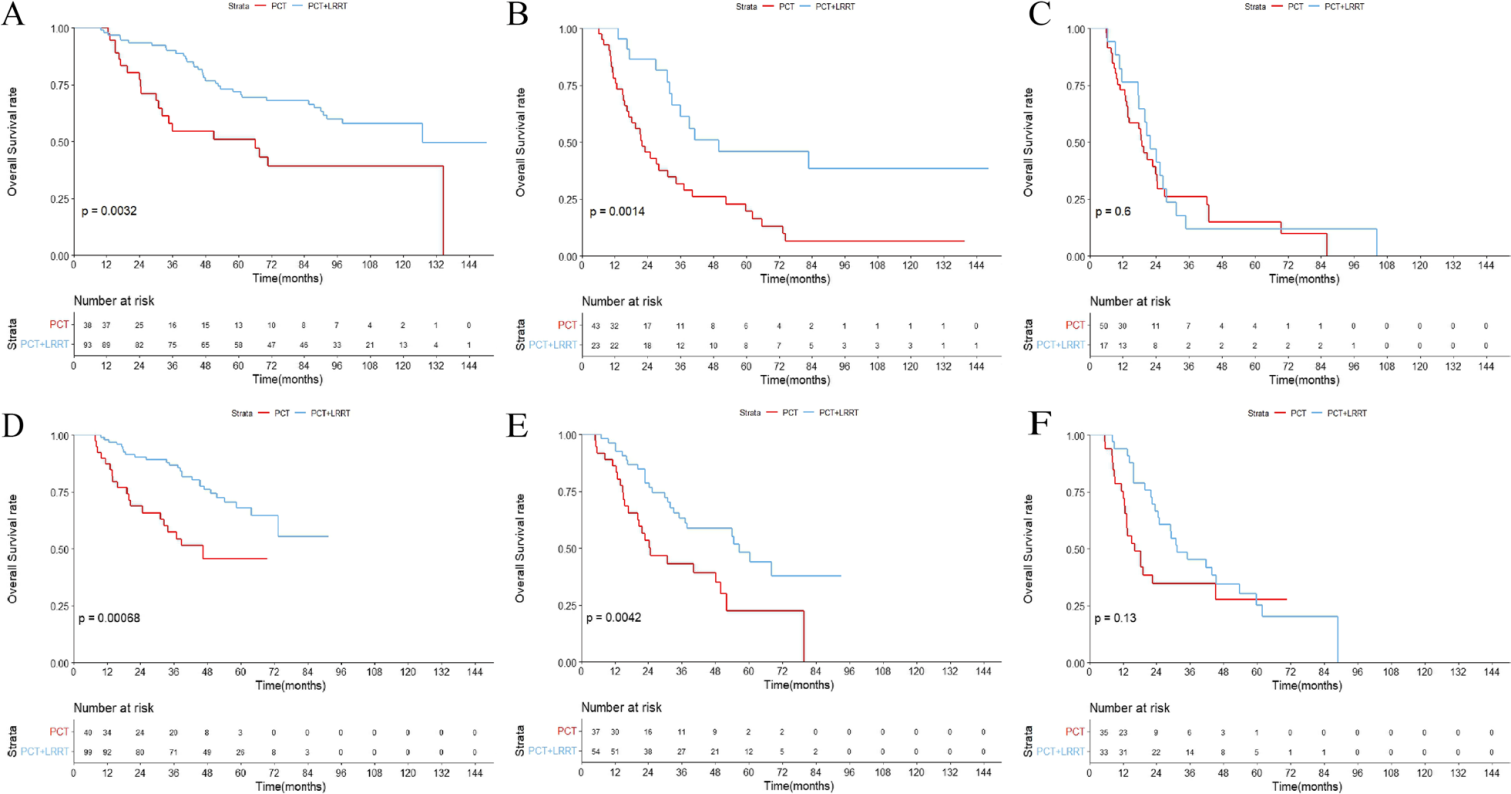
Comparison of overall survival for palliative chemotherapy (PCT) alone versus PCT + locoregional intensity-modulated radiotherapy (LRRT) in the training cohort: low-risk group (**A**); intermediate-risk group (**B**); high-risk group (**C**). Comparison of overall survival for PCT alone versus PCT + LRRT in the training cohort: low-risk group (**D**); intermediate-risk group (**E**); high-risk group (**F**)

We carried out subgroup analyses for OS with PCT alone versus PCT followed by LRRT in the different risk groups. A total of 131 (49.6%) patients received PCT alone, and 133 (50.4%) received PCT followed by LRRT in the training group, while 112 (37.6%) and 186 (62.4%) in the validation group, respectively. In the low-risk group, the 3-year OS rate in patients treated with PCT followed by LRRT was significantly higher than in patients only treated with PCT alone (89.9% vs. 54.5%, *P* = 0.0032), and the same result was observed in the intermediate-risk group (61.2% vs. 31.7%, *P* = 0.0014). However, we did not observe significant survival differences in the high-risk group (11.7% vs. 26.1%, *P* = 0.6) (Fig. 4A–C). We then performed separate multivariate analyses to determine whether the efficacy of additional LRRT was an independent prognostic factor for OS in these three risk groups. LRRT remained an independent prognostic factor after adjusting for number of involved lesions, liver involvement, response statues to PCT, age, and EBV-DNA in the low- and intermediate-risk groups (low-risk: adjusted HR: 0.468; 95% CI: 0.263–0.831; *P* = 0.010; intermediate-risk: adjusted HR: 0.412; 95% CI, 0.220–0.772; *P* = 0.006). However, LRRT was not associated with OS in the high-risk group (adjusted HR: 1.211; 95% CI, 0.586–2.506; *P* = 0.605) (Table 3). The clinical characteristics of the different risk groups are presented in Additional file 1: Table S1. In the validation group, the 3-year OS rate was also higher in patients treated with PCT followed by LRRT than in those treated with PCT alone in both the low- and intermediate-risk groups (*P* < 0.05 for all), but no significant difference was observed in the high-risk group (*P* = 0.13) (Fig. 4D–F). Similarly, LRRT was also significantly associated with OS on separate multivariable analyses (low-risk: adjusted HR: 0.337; 95% CI: 0.183–0.621; *P* = 0.001; intermediate-risk: adjusted HR: 0.445; 95% CI, 0.252–0.786; *P* = 0.005), but not in the high-risk group (adjusted HR: 0.721; 95% CI, 0.389–1.333; *P* = 0.297) (Table 3). Additional file 2: Table S2 summarizes the characteristics of different risk groups in the validation cohort.

**Table 3:** Multivariable analysis of overall survival by treatment modality in different risk groupings

Treatment strategy	Low risk	*P* value	Intermediate risk	*P* value	High risk	*P* value
	Adjusted HR (95% CI)		Adjusted HR (95% CI)		Adjusted HR (95% CI)	
PCT + LRRT versus PCT alone						
Training Cohort	0.468 (0.236–0.831)	0.010	0.412 (0.220–0.772)	0.006	1.211 (0.586–2.506)	0.605
Validation Cohort	0.337 (0.183–0.621)	0.001	0.445 (0.252–0.786)	0.005	0.721 (0.389–0.1.333)	0.297

*PCT* palliative chemotherapy, *LRRT* locoregional intensity-modulated radiotherapy, *EBV* Epstein–Barr virus, *HR* hazard ratio, *CI* confidence interval

Multivariable Cox regression model adjusted for number of involved lesions, liver involvement, response statues to PCT, age, EBV-DNA and treatment modality

### Clinical outcome of concurrent chemoradiotherapy

**Fig. 5:**
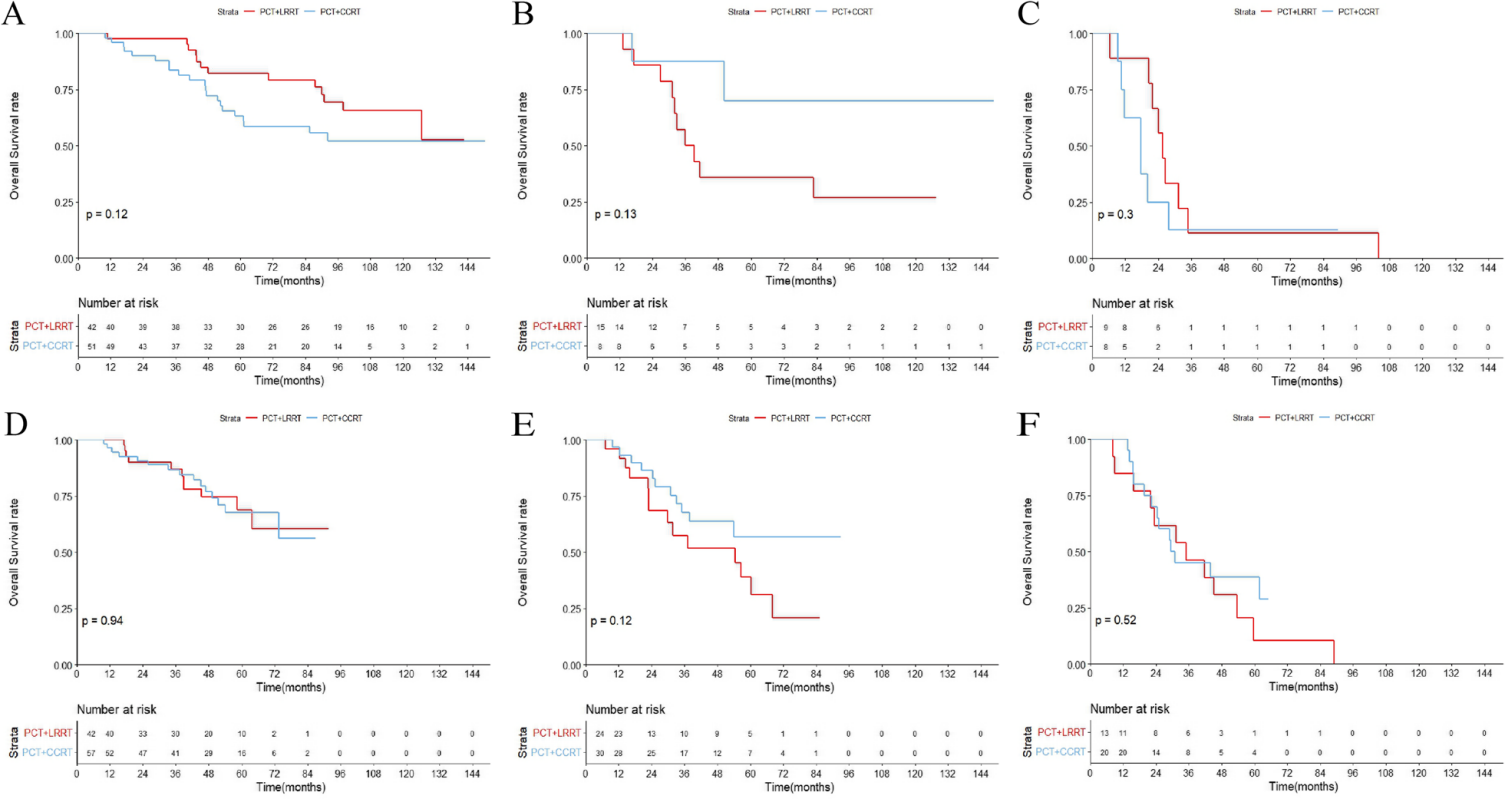
Comparison of overall survival for palliative chemotherapy (PCT) + locoregional intensity-modulated radiotherapy (LRRT) alone versus PCT + concurrent chemoradiotherapy (CRRT) in the training cohort: low-risk group (**A**); intermediate-risk group (**B**); high-risk group (**C**). Comparison of overall survival for PCT + LRRT alone versus PCT + CRRT in the training cohort: low-risk group (**D**); intermediate-risk group (**E**); high-risk group (**F**)

In the training group, 66 (49.6%) patients were treated with PCT followed by LRRT alone, while 67 (50.4%) received CCT during LRRT. The median CCT cycle was 2. No significant differences in OS were observed between these two patterns of radiotherapy, the 3-year OS rates in the LRRT alone and concurrent chemoradiotherapy (CCRT) groups were 74.6% and 75.1%, respectively (*P* = 0.88). In the subgroup analysis, the CCRT group still had no survival benefit over the LRRT group in these three groups (*P* = 0.12, *P* = 0.13, and *P* = 0.3, respectively) (Fig. 5A–C). Clinical characteristics of the patients are shown in Additional file 3: Table S3. In the validation group, 79 (42.5%) patients were treated with PCT followed by LRRT alone, while 107 (57.5%) received CCT during LRRT. There were also no significant clinical disparities with the validation cohort (3-year OS rate: 70.9% vs. 72.9%, *P* = 0.21) or in the three risk groups (*P* = 0.94, *P* = 0.12, and *P* = 0.52, respectively) (Fig. 5D–F). Clinical characteristics of the validation cohort are listed in Additional file 4: Table S4.

## Discussion

Due to the complexity and heterogeneity of dmNPC, the AJCC TNM staging system is unable to subgroup those patients and provide practical guidance for individualized treatment. To better risk stratify dmNPC and realize the potential value of additional LRRT, we divided patients into three groups with significantly different clinical outcomes through the autoRPA web server. This study provided new insights into risk stratification for dmNPC and demonstrated that additional LRRT significantly prolonged OS in the low- and intermediate-risk groups. CCT did not appear to improve OS in dmNPC during radiotherapy in our study.

Although definite radiotherapy represents a curative therapeutic modality in non-metastatic NPC, there is little consensus on how to clarify which dmNPC benefits would benefit from it [12]. Our previous research demonstrated that the long-term prognosis of dmNPC was directly influenced by distant metastatic status, which engendered a significant challenge for devising optimal treatment strategies [13]. Chen et al. reported that dmNPC with liver involvement irrespective of the presence of metastatic lesions had the worst survival and could not benefit from additional LRRT [14]. However, this subdivision of dmNPC did not include plasma EBV-DNA, which is strongly linked to the prognosis and prediction of the treatment response. Sun et al. divided metachronous and synchronous metastatic patients into four groups with distinct OS by incorporating EBV-DNA copy number, liver metastatic status, and the metastatic status [15]. They revealed that a high EBV-DNA copy number (≥ 33,000 copies/mL) and liver invasion were the worst prognostic factors for OS. However, that study did not provide a standard for stratifying dmNPC and guiding treatment strategies, and accurate risk assessment models are significantly needed. Our study conducted a comprehensive multivariable analysis and showed that poor prognostic factors included age larger than 52, high copy number of pretreated EBV-DNA, liver involvement, unsatisfactory tumor response to PCT (SD/PD) and more than 4 involved lesions. These five risk factors were analyzed through the web server autoRPA for cancer staging, and the number of involved lesions, liver involvement, and pretreated EBV-DNA copy number were finally used to form a novel prediction model. Thus, autoRPA represents an efficient, reliable, and easily applicable method for constructing prognostic models for risk stratification.

Unlike previous studies, our model combined both anatomical and biological factors, and the optimal factors were selected to risk stratify patients accurately [16, 17]. Based on our model, dmNPC patients were divided into three risk categories with significant differences in OS: the low-risk group (involved lesions ≤ 4 without liver involvement), the intermediate-risk group (involved lesions ≤ 4 with liver involvement or involved lesions > 4 with EBV-DNA < 62,000 copies/ml) and the high-risk group (involved lesions > 4 with EBV-DNA > 62,000 copies/ml). The number of involved lesions was the most significant risk factor, and patients in the low-risk group had the best prognosis, with a 3-year OS rate significantly higher than that in the intermediate-risk and high-risk groups [15, 18]. Perhaps the most straightforward explanation is that multiple involved lesions reflect a greater tumor burden and higher EBV-DNA burden. Bone was the most common site of metastasis in our research; however, it was not a significant factor related to prognosis. Liver metastasis was shown to be a critical prognostic factor, which may be explained that patients with liver metastases may have impaired liver function, resulting in poor therapy tolerability. Moreover, plasma EBV-DNA is an essential indicator of the tumor burden of NPC, which has been proposed for tumor staging and monitoring treatment response and is an adverse prognostic factor [19, 20]. Some studies have revealed that pretreatment plasma EBV-DNA combined with TNM staging could be used to derive a better model for predicting nonmetastatic NPC patient survival [3]. However, the concentration of plasma-pretreated EBV-DNA on grouping dmNPC was rare, and it was insufficient for dividing EBV-DNA into negative and positive for grouping. Therefore, our model successfully divided patients into various risk groups with a cutoff value of 62,000 copies/ml, which may aid in risk stratification.

The prospective study reported by Chen et al. showed that PCT followed by LRRT provided a significant OS benefit as well as improved progression-free survival (PFS) in dmNPC [5]. Similar to that study, patients stratified into the low- and intermediate-risk groups benefited from additional LRRT, whereas patients in the high-risk group did not. Several studies have shown that dmNPC with a high tumor burden is unlikely to benefit from additional LRRT [21–23]. This could be explained that patients stratified in the low- and intermediate-risk group were in the low tumor burden status, and distant metastases could be better controlled under intensive PCT. In this study, the clinical characteristics, and responses to PCT were statistically balanced between the PCT group and the non-radiotherapy group, demonstrating that additional LRRT reduces the rate of local recurrence after PCT in patients with low tumor burden status. Therefore, LRRT becomes a critical factor in prolonging OS in these patients. Due to the rapid progression, an unconsidered response rate to PCT was observed in the high-risk group, and treatment failures of distant metastases were the major cause of death. We speculated that the microenvironment in patients with multiple involved lesions and a high copy number of pretreated EBV-DNA was heavily immunosuppressive. In addition, an immunosuppressive microenvironment could rapidly facilitate disease progression and hamper antitumor therapy [24, 25]. Therefore, the effect of additional LRRT would be dismissed in patients with a high tumor burden, let alone it resulting in inevitable toxicity such as hearing loss and brain damage. Recently, much progress has been made in immunotherapy for treating recurrent or metastatic NPC. Programmed death-1 inhibitors in combination with chemotherapy have shown promising outcomes compared with chemotherapy alone and an acceptable safety profile, with manageable toxicities [26, 27].

Compared with radiotherapy alone, CCRT improved the tumor response of non-metastatic advanced NPC, as well as the 5-year OS and PFS rates, in a previous study [28]. However, there is still no consensus on whether CCRT leads to survival benefits in dmNPC. In our study, PCT followed by CCRT did not improve OS over PCT followed by LRRT alone, which is consistent with a previous report [29]. Different from the treatment regimen for the locally advanced patients, over 90% of the patients (294 out of 319) in this study had received 4 or more cycles of platinum-based PCT before LRRT, and the ORR to PCT was 75.9% for LRRT alone cohort and 69.5% for CCRT cohort (*P* = 0.208). It indicated that dmNPC patients are sufficiently treated with 4 or more cycles of intensive PCT. Moreover, CCRT was associated with acute toxicity, such as chemoradiotherapy-induced myelosuppression, significantly decreasing patient compliance and tolerance to LRRT. Our findings suggest that further evaluation of platinum-based CCT as a potential radiosensitizer for dmNPC is warranted.

There were several limitations in our study. First, the study was conducted at a single cancer center in an endemic area that lacked pathological diversity. Second, the study was retrospective in nature, and selection bias must be considered. Third, although all the palliative treatments were platinum-based chemotherapy, the first-line regimens were not uniform. Finally, although our model had an extremely high performance in predicting the prognosis of dmNPC, well-designed prospective clinical trials are warranted to confirm our findings.

## Conclusions

In conclusion, our novel RPA model effectively stratified dmNPC into three risk groups with significantly different survival outcomes. The application of this model could help facilitate treatment recommendations for adding LRRT, and the addition of CCT to LRRT did not result in survival benefits in dmNPC patients.

## Supplementary Information

**Additional file 1: Table S1** Clinical characteristics of different risk groups in the training cohort.

**Additional file 2: Table S2** Clinical characteristics of different risk groups in the validation cohort.

**Additional file 3: Table S3** Clincial characteristic of the patients treated with or without CCT during LRRT in the training cohort.

**Additional file 4: Table S4** Clincial characteristic of the patients treated with or without CCT during LRRT in the validation cohort.

## Data Availability

The datasets analysed during this study are available from the authors on reasonable request.

## Abbreviations

NPC: Nasopharyngeal carcinoma
dmNPC: De novo metastatic nasopharyngeal carcinoma
TNM: Tumor-node-metastasis
LRRT: Locoregional intensity-modulated radiotherapy
PCT: Palliative chemotherapy
IMRT: Intensity-modulated radiotherapy
CCT: Concurrent chemotherapy
CCRT: Concurrent chemoradiotherapy
EBV: Epstein–Barr virus
RPA: Recursive partitioning analysis
OS: Overall survival
ROC: Receiver operating characteristic
AUROC: Area under ROC
CI: Confidence interval
CR: Complete remission
PR: Partial response
PD: Progressive disease
SD: Stable disease
ECOG: Eastern Cooperative Oncology Group
CT: Computed tomography
MRI: Magnetic resonance imaging
ECT: Emission computed tomography
PET: Positron emission tomography
AJCC: American Joint Committee on Cancer
SYSUCC: Sun Yat-sen University Cancer Center
