# Supplementary material for: Proposed prognostic subgroups and facilitated clinical decision-making for additional locoregional radiotherapy in de novo metastatic nasopharyngeal carcinoma: a retrospective study based on recursive partitioning analysis
Source: Radiat Oncol. 2023 Jan 21;18:15. doi: 10.1186/s13014-022-02168-2 (PMC9862810; doi:10.1186/s13014-022-02168-2)
Supplement: Supplementary file 1 — Additional file 1: Table S1 Clinical characteristics of different risk groups in the training cohort. [file 13014_2022_2168_MOESM1_ESM.docx]

**Table S1 Clinical characteristics of different risk groups in the training cohort**

|  | **Low risk** | |  | **Intermediate risk** | |  | **High risk** | |  |
| --- | --- | --- | --- | --- | --- | --- | --- | --- | --- |
|  | **PCT**  **No. (%)** | **PCT+LRRT**  **No. (%)** | **P** | **PCT**  **No. (%)** | **PCT+LRRT**  **No. (%)** | **P** | **PCT**  **No. (%)** | **PCT+LRRT**  **No. (%)** | **P** |
| **Total** | 38 | 93 |  | 43 | 23 |  | 50 | 17 |  |
| **Age(years)** |  |  |  |  |  |  |  |  |  |
| ≤ 52 | 22 (57.9) | 69 (74.2) | 0.066 | 26 (60.5) | 17 (73.9) | 0.275 | 35 (70.0) | 10 (58.8) | 0.397 |
| > 52 | 16 (42.1) | 24 (25.8) |  | 17 (39.5) | 6 (26.1) |  | 15 (30.0) | 7 (41.2) |  |
| **Sex** |  |  |  |  |  |  |  |  |  |
| Male | 30 (78.9) | 78 (83.9) | 0.501 | 36 (83.7) | 15 (65.2) | 0.087 | 45 (90.0) | 14 (82.4) | 0.684 |
| Female | 8 (21.1) | 15 (16.1) |  | 7 (16.3) | 8 (34.8) |  | 5 (10.0) | 3 (17.6) |  |
| **Famlily history of NPC** |  |  |  |  |  |  |  |  |  |
| No | 35 (92.1) | 87 (93.5) | 1.000 | 43 (100.0) | 21 (91.3) | 0.118 | 44 (88.0) | 15 (88.2) | 1.000 |
| Yes | 3 (7.9) | 6 (6.5) |  | 0 | 2 (8.7) |  | 6 (12.0) | 2 (11.8) |  |
| **Smoking history** |  |  |  |  |  |  |  |  |  |
| No | 17 (44.7) | 59 (63.4) | 0.049 | 25 (58.1) | 17 (73.9) | 0.204 | 28 (56.0) | 11 (64.7) | 0.530 |
| Yes | 21 (55.3) | 34 (36.6) |  | 18 (41.9) | 6 (26.1) |  | 22 (44.0) | 6 (35.3) |  |
| **Drinking history** |  |  |  |  |  |  |  |  |  |
| No | 33 (86.8) | 83 (89.2) | 0.695 | 37 (86.0) | 22 (95.7) | 0.431 | 48 (96.0) | 16 (94.1) | 1.000 |
| Yes | 5 (13.2) | 10 (10.8) |  | 6 (14.0) | 1 (4.3) |  | 2 (4.0) | 1 (5.9) |  |
| **Tumor stage*** |  |  |  |  |  |  |  |  |  |
| T1-T2 | 5 (13.2) | 18 (19.4) | 0.398 | 8 (18.6) | 2 (8.7) | 0.478 | 7 (14.0) | 2 (11.8) | 1.000 |
| T3-T4 | 33 (86.8) | 75 (80.6) |  | 35 (81.4) | 21 (91.3) |  | 43 (86.0) | 15 (88.2) |  |
| **Node stage*** |  |  |  |  |  |  |  |  |  |
| N0-N1 | 6 (15.8) | 21 (22.6) | 0.383 | 8 (18.6) | 5 (21.7) | 0.760 | 6 (12.0) | 3 (17.6) | 0.859 |
| N2-N3 | 32 (84.2) | 72 (77.4) |  | 35 (81.4) | 18 (78.3) |  | 44 (88.0) | 14 (82.4) |  |
| **Bone involvement** |  |  |  |  |  |  |  |  |  |
| No | 15 (39.5) | 27 (29.0) | 0.245 | 19 (44.2) | 11 (47.8) | 0.777 | 11 (22.0) | 0 | 0.053 |
| Yes | 23 (60.5) | 66 (71.0) |  | 24 (55.8) | 12 (52.2) |  | 39 (78.0) | 17 (100.0) |  |
| **Lung involvement** |  |  |  |  |  |  |  |  |  |
| No | 25 (65.8) | 69 (74.2) | 0.332 | 36 (83.7) | 20 (87.0) | 1.000 | 36 (72.0) | 14 (82.4) | 0.600 |
| Yes | 13 (34.2) | 24 (25.8) |  | 7 (16.3) | 3 (13.0) |  | 14 (28.0) | 3 (17.6) |  |
| **Liver involvement** |  |  |  |  |  |  |  |  |  |
| No | 38 (100.0) | 93 (100.0) | NA | 14 (32.6) | 9 (39.1) | 0.593 | 18 (36.0) | 13 (76.5) | 0.009 |
| Yes | 0 | 0 |  | 29 (67.4) | 14 (60.9) |  | 32 (64.0) | 4 (23.5) |  |
| **Distant lymph node involvement** |  |  |  |  |  |  |  |  |  |
| No | 36 (94.7) | 82 (88.2) | 0.413 | 37 (86.0) | 22 (95.7) | 0.431 | 30 (60.0) | 13 (76.5) | 0.352 |
| Yes | 2 (5.3) | 11 (11.8) |  | 6 (14.0) | 1 (4.3) |  | 20 (40.0) | 4 (23.5) |  |
| **Number of involved organs** |  |  |  |  |  |  |  |  |  |
| Single | 38 (100.0) | 87 (93.5) | 0.180 | 28 (65.1) | 17 (73.9) | 0.465 | 13 (26.0) | 9 (52.9) | 0.041 |
| Multiple | 0 | 6 (6.5) |  | 15 (34.9) | 6 (26.1) |  | 37 (74.0) | 8 (47.1) |  |
| **Number of involved lesions** |  |  |  |  |  |  |  |  |  |
| ≤ 4 | 38 (100.0) | 93 (100.0) | NA | 22 (51.2) | 13 (56.5) | 0.678 | 0 | 0 | NA |
| > 4 | 0 | 0 |  | 21 (48.8) | 10 (43.5) |  | 50 (100.0) | 17 (100.0) |  |
| **EBV-DNA status** |  |  |  |  |  |  |  |  |  |
| EBV-DNA ≤ 62000 | 22 (57.9) | 66 (71.0) | 0.148 | 30 (69.8) | 17 (73.9) | 0.723 | 0 | 0 | NA |
| EBV-DNA > 62000 | 16 (42.1) | 27 (29.0) |  | 13 (30.2) | 6 (26.1) |  | 50 (100.0) | 17 (100.0) |  |
| **Chemotherapy cycle** |  |  |  |  |  |  |  |  |  |
| < 4 | 3 (7.9) | 13 (14.0) | 0.502 | 5 (11.6) | 1 (4.3) | 0.595 | 4 (8.0) | 1 (5.9) | 1.000 |
| ≥ 4 | 35 (92.1) | 80 (86.0) |  | 38 (88.4) | 22 (95.7) |  | 46 (92.0) | 16 (94.1) |  |
| **Tumor response to PCT** |  |  |  |  |  |  |  |  |  |
| PR/CR | 28 (73.7) | 75 (80.6) | 0.378 | 23 (53.5) | 17 (73.9) | 0.106 | 19 (38.0) | 10 (58.8) | 0.134 |
| SD/PD | 10 (26.3) | 18 (19.4) |  | 20 (46.5) | 6 (26.1) |  | 31 (62.0) | 7 (41.2) |  |

*NPC* nasopharyngeal carcinoma, *PCT* palliative chemotherapy, *IMRT* intensity-modulated radiotherapy, *LRRT* locoregional intensity-modulated radiotherapy, *CCT* concurrent chemotherapy, *EBV* Epstein–Barr virus, *No.* Number, *NA* not applicable, *CR* complete response, *PR* partial response, *PD* disease progression, *SD* stable disease.

*According to the 8th TNM staging system.
