# Supplementary material for: Proposed prognostic subgroups and facilitated clinical decision-making for additional locoregional radiotherapy in de novo metastatic nasopharyngeal carcinoma: a retrospective study based on recursive partitioning analysis
Source: Radiat Oncol. 2023 Jan 21;18:15. doi: 10.1186/s13014-022-02168-2 (PMC9862810; doi:10.1186/s13014-022-02168-2)
Supplement: Supplementary file 2 — Additional file 2: Table S2 Clinical characteristics of different risk groups in the validation cohort. [file 13014_2022_2168_MOESM2_ESM.docx]

**Table S2 Clinical characteristics of different risk groups in the validation cohort**

|  | **Low risk** | |  | **Intermediate risk** | |  | **High risk** | |  |
| --- | --- | --- | --- | --- | --- | --- | --- | --- | --- |
|  | **PCT**  **No. (%)** | **PCT+LRRT**  **No. (%)** | **P** | **PCT**  **No. (%)** | **PCT+LRRT**  **No. (%)** | **P** | **PCT**  **No. (%)** | **PCT+LRRT**  **No. (%)** | **P** |
| **Total** | 40 | 99 |  | 37 | 54 |  | 35 | 33 |  |
| **Age(years)** |  |  |  |  |  |  |  |  |  |
| ≤ 52 | 31 (77.5) | 72 (72.7) | 0.561 | 24 (64.9) | 33 (61.1) | 0.716 | 23 (65.7) | 27 (81.8) | 0.132 |
| 52 | 9 (22.5) | 27 (27.3) |  | 13 (35.1) | 21 (38.9) |  | 12 (34.3) | 6 (18.2) |  |
| **Sex** |  |  |  |  |  |  |  |  |  |
| Male | 30 (75.0) | 82 (82.8) | 0.291 | 33 (89.2) | 46 (85.2) | 0.811 | 32 (91.4) | 29 (87.9) | 0.705 |
| Female | 10 (25.0) | 17 (17.2) |  | 4 (10.8) | 8 (14.8) |  | 3 (8.6) | 4 (12.1) |  |
| **Famlily history of NPC** |  |  |  |  |  |  |  |  |  |
| No | 38 (95.0) | 95 (96.0) | 1.000 | 35 (94.6) | 47 (87.0) | 0.407 | 30 (85.7) | 32 (97.0) | 0.227 |
| Yes | 2 (5.0) | 4 (4.0) |  | 2 (5.4) | 7 (13.0) |  | 5 (14.3) | 1 (3.0) |  |
| **Smoking history** |  |  |  |  |  |  |  |  |  |
| No | 27 (67.5) | 70 (70.7) | 0.709 | 19 (51.4) | 32 (59.3) | 0.455 | 21 (60.0) | 22 (66.7) | 0.569 |
| Yes | 13 (32.5) | 29 (29.3) |  | 18 (48.6) | 22 (40.7) |  | 14 (40.0) | 11 (33.3) |  |
| **Drinking history** |  |  |  |  |  |  |  |  |  |
| No | 37 (92.5) | 95 (96.0) | 0.410 | 35 (94.6) | 49 (90.7) | 0.782 | 32 (91.4) | 30 (90.9) | 1.000 |
| Yes | 3 (7.5) | 4 (4.0) |  | 2 (5.4) | 5 (9.3) |  | 3 (8.6) | 3 (9.1) |  |
| **Tumor stage*** |  |  |  |  |  |  |  |  |  |
| T1-T2 | 2 (5.0) | 12 (12.1) | 0.341 | 5 (13.5) | 4 (7.4) | 0.548 | 4 (11.4) | 4 (12.1) | 1.000 |
| T3-T4 | 38 (95.0) | 87 (87.9) |  | 32 (86.5) | 50 (92.6) |  | 31 (88.6) | 29 (87.9) |  |
| **Node stage*** |  |  |  |  |  |  |  |  |  |
| N0-N1 | 4 (10.0) | 18 (18.2) | 0.232 | 4 (10.8) | 12 (22.2) | 0.261 | 3 (8.6) | 1 (3.0) | 0.614 |
| N2-N3 | 36 (90.0) | 81 (81.8) |  | 33 (89.2) | 42 (77.8) |  | 32 (91.4) | 32 (97.0) |  |
| **Bone involvement** |  |  |  |  |  |  |  |  |  |
| No | 9 (22.5) | 28 (28.3) | 0.485 | 11 (29.7) | 23 (42.6) | 0.213 | 6 (17.1) | 5 (15.2) | 0.824 |
| Yes | 31 (77.5) | 71 (71.7) |  | 26 (70.3) | 31 (57.4) |  | 29 (82.9) | 28 (84.8) |  |
| **Lung involvement** |  |  |  |  |  |  |  |  |  |
| No | 30 (75.0) | 78 (78.8) | 0.627 | 31 (83.8) | 42 (77.8) | 0.480 | 20 (57.1) | 23 (69.7) | 0.283 |
| Yes | 10 (25.0) | 21 (21.2) |  | 6 (16.2) | 12 (22.2) |  | 15 (42.9) | 10 (30.3) |  |
| **Liver involvement** |  |  |  |  |  |  |  |  |  |
| No | 40 (100.0) | 99 (100.0) | NA | 20 (54.1) | 24 (44.4) | 0.368 | 17 (48.6) | 23 (69.7) | 0.077 |
| Yes | 0 | 0 |  | 17 (45.9) | 30 (55.6) |  | 18 (51.4) | 10 (30.3) |  |
| **Distant lymph node involvement** |  |  |  |  |  |  |  |  |  |
| No | 35 (87.5) | 84 (84.8) | 0.687 | 26 (70.3) | 47 (87.0) | 0.050 | 16 (45.7) | 22 (66.7) | 0.082 |
| Yes | 5 (12.5) | 15 (15.2) |  | 11 (29.7) | 7 (13.0) |  | 19 (54.3) | 11 (33.3) |  |
| **Number of involved organs** |  |  |  |  |  |  |  |  |  |
| Single | 34 (85.0) | 90 (90.9) | 0.309 | 21 (56.8) | 33 (61.1) | 0.678 | 6 (17.1) | 15 (45.5) | 0.012 |
| Multiple | 6 (15.0) | 9 (9.1) |  | 16 (43.2) | 21 (38.9) |  | 29 (82.9) | 18 (54.5) |  |
| **Number of involved lesions** |  |  |  |  |  |  |  |  |  |
| ≤ 4 | 40 (100.0) | 99 (100.0) | NA | 10 (27.0) | 23 (42.6) | 0.129 | 0 | 0 | NA |
| > 4 | 0 | 0 |  | 27 (73.0) | 31 (57.4) |  | 35 (100.0) | 31 (100.0) |  |
| **EBV-DNA status (copies/ml)** |  |  |  |  |  |  |  |  |  |
| EBV-DNA < 62000 | 32 (80.0) | 76 (76.8) | 0.679 | 33 (89.2) | 49 (90.7) | 1.000 | 0 | 0 | NA |
| EBV-DNA > 62000 | 8 (20.0) | 23 (23.3) |  | 4 (10.8) | 5 (9.3) |  | 35 (100.0) | 33 (100.0) |  |
| **Tumor response to PCT** |  |  |  |  |  |  |  |  |  |
| PR/CR | 23 (57.5) | 70 (70.7) | 0.134 | 27 (73.0) | 42 (77.8) | 0.599 | 12 (34.3) | 17 (51.5) | 0.151 |
| PD/SD | 17 (42.5) | 29 (29.3) |  | 10 (27.0) | 12 (22.2) |  | 23 (65.7) | 16 (48.5) |  |
| **Chemotherapy cycle** |  |  |  |  |  |  |  |  |  |
| < 4 | 0 | 8 (8.1) | 0.105 | 2 (5.4) | 1 (1.9) | 0.564 | 7 (20.0) | 1 (3.0) | 0.073 |
| ≥ 4 | 40 (100.0) | 91 (91.9) |  | 35 (94.6) | 53(98.1) |  | 28 (80.0) | 32 (97.0) |  |

*NPC* nasopharyngeal carcinoma, *PCT* palliative chemotherapy, *IMRT* intensity-modulated radiotherapy, *LRRT* locoregional intensity-modulated radiotherapy, *CCT* concurrent chemotherapy, *EBV* Epstein–Barr virus, *No.* Number, *NA* not applicable, *CR* complete response, *PR* partial response, *PD* disease progression, *SD* stable disease.

*According to the 8th TNM staging system.
