# Supplementary material for: Proposed prognostic subgroups and facilitated clinical decision-making for additional locoregional radiotherapy in de novo metastatic nasopharyngeal carcinoma: a retrospective study based on recursive partitioning analysis
Source: Radiat Oncol. 2023 Jan 21;18:15. doi: 10.1186/s13014-022-02168-2 (PMC9862810; doi:10.1186/s13014-022-02168-2)
Supplement: Supplementary file 3 — Additional file 3: Table S3 Clincial characteristic of the patients treated with or without CCT during LRRT in the training cohort. [file 13014_2022_2168_MOESM3_ESM.docx]

**Table S3 Clincial characteristic of the patients treated with or without CCT during LRRT in the training cohort**

|  | **Low-risk group** | |  | **Intermediate -risk group** | |  | **High-risk group** | |  |
| --- | --- | --- | --- | --- | --- | --- | --- | --- | --- |
| **Pattern of Radiotherapy** | **PCT+LRRT**  **No. (%)** | **PCT+CCRT**  **No. (%)** |  | **PCT+LRRT**  **No. (%)** | **PCT+CCRT**  **No. (%)** |  | **PCT+LRRT**  **No. (%)** | **PCT+CCRT**  **No. (%)** |  |
| **Total** | 42 | 51 |  | 15 | 8 |  | 9 | 8 |  |
| **Bone involvement** |  |  |  |  |  |  |  |  |  |
| No | 14 (33.3) | 13 (25.5) | 0.407 | 7 (46.7) | 4 (50.0) | 1.000 | 0 | 0 | NA |
| Yes | 28 (66.7) | 38 (74.5) |  | 8 (53.3) | 4 (50.0) |  | 9 (100.0) | 8 (100.0) |  |
| **Lung involvement** |  |  |  |  |  |  |  |  |  |
| No | 29 (69.0) | 40 (78.4) | 0.303 | 13 (86.7) | 7 (87.5) | 1.000 | 9 (100.0) | 5 (62.5) | 0.082 |
| Yes | 13 (31.0) | 11 (21.6) |  | 2 (13.3) | 1 (12.5) |  | 0 | 3 (37.5) |  |
| **Liver involvement** |  |  |  |  |  |  |  |  |  |
| No | 42 (100.0) | 51 (100.0) | NA | 6 (40.0) | 3 (37.5) | 1.000 | 5 (55.6) | 8 (100.0) | 0.082 |
| Yes | 0 | 0 |  | 9 (60.0) | 5 (62.5) |  | 4 (44.4) | 0 |  |
| **Distant lymph node involvement** |  |  |  |  |  |  |  |  |  |
| No | 40 (95.2) | 42 (82.4) | 0.111 | 15 (100.0) | 7 (87.5) | 0.348 | 7 (77.8) | 6 (75.0) | 1.000 |
| Yes | 2 (4.8) | 9 (17.6) |  | 0 | 1 (12.5) |  | 2 (22.2) | 2 (25.0) |  |
| **Number of involved organs** |  |  |  |  |  |  |  |  |  |
| Single | 41 (97.6) | 46 (90.2) | 0.305 | 11 (73.3) | 6 (75.0) | 1.000 | 5 (55.6) | 4 (50.0) | 1.000 |
| Multiple | 1 (2.4) | 5 (9.8) |  | 4 (26.7) | 2 (25.0) |  | 4 (44.4) | 4 (50.0) |  |
| **Number of involved lesions** |  |  |  |  |  |  |  |  |  |
| ≤ 4 | 42 (100.0) | 51 (100.0) | NA | 8 (53.3) | 5 (62.5) | 1.000 | 0 | 0 | NA |
| > 4 | 0 | 0 |  | 7 (46.7) | 3 (37.5) |  | 9 (100.0) | 8 (100.0) |  |
| **EBV-DNA status (copies/ml)** |  |  |  |  |  |  |  |  |  |
| EBV-DNA ≤ 62000 | 28 (66.7) | 38 (74.5) | 0.407 | 12 (80.0) | 5 (62.5) | 0.621 | 0 | 0 | NA |
| EBV-DNA >62000 | 14 (33.3) | 13 (25.5) |  | 3 (20.0) | 3 (37.5) |  | 9 (100.0) | 8 100.0() |  |
| **Chemotherapy cycle** |  |  |  |  |  |  |  |  |  |
| < 4 | 3 (7.1) | 10 (19.6) | 0.154 | 0 | 1 (12.5) | 0.348 | 0 | 1 (12.5) | 0.471 |
| ≥ 4 | 39 (92.9) | 41 (80.4) |  | 15 (100.0) | 7 (87.5) |  | 9 (100.0) | 7 (87.5) |  |
| **Tumor response to PCT** |  |  |  |  |  |  |  |  |  |
| PR/CR | 34 (81.0) | 41 (80.4) | 0.946 | 12 (80.0) | 5 (62.5) | 0.621 | 7 (77.8) | 3 (37.5) | 0.153 |
| PD/SD | 8 (19.0) | 10 (19.6) |  | 3 (20.0) | 3 (37.5) |  | 2 (22.2) | 5 (62.5) |  |
| **Radiotherapy dose of loco-regional** |  |  |  |  |  |  |  |  |  |
| ＜66Gy | 0 | 1 (2.0) | 1.000 | 0 | 0 | NA | 0 | 0 | NA |
| ≥66Gy | 42 (100.0) | 50 (98.0) |  | 15 (100.0) | 8 (100.0) |  | 9 (100.0) | 8 (100.0) |  |

*NPC* nasopharyngeal carcinoma, *PCT* palliative chemotherapy, *LRRT* locoregional intensity-modulated radiotherapy, *CCT* concurrent chemotherapy, *CCRT* concurrent chemoradiotherapy, *EBV* Epstein–Barr virus, *No.* Number, *NA* not applicable, *CR* complete response, *PR* partial response, *PD* disease progression, *SD* stable disease.

*According to the 8th TNM staging system.
