# Supplementary material for: Proposed prognostic subgroups and facilitated clinical decision-making for additional locoregional radiotherapy in de novo metastatic nasopharyngeal carcinoma: a retrospective study based on recursive partitioning analysis
Source: Radiat Oncol. 2023 Jan 21;18:15. doi: 10.1186/s13014-022-02168-2 (PMC9862810; doi:10.1186/s13014-022-02168-2)
Supplement: Supplementary file 4 — Additional file 4: Table S4 Clincial characteristic of the patients treated with or without CCT during LRRT in the validation cohort. [file 13014_2022_2168_MOESM4_ESM.docx]

**Table S4 Clincial characteristic of the patients treated with or without CCT during LRRT in the validation cohort.**

|  | **Low-risk group** | |  | **Intermediate -risk group** | |  | **High-risk group** | |  |
| --- | --- | --- | --- | --- | --- | --- | --- | --- | --- |
|  | **PCT+LRRT**  **No. (%)** | **PCT+CCRT**  **No. (%)** | **P** | **PCT+LRRT**  **No. (%)** | **PCT+CCRT**  **No. (%)** | **P** | **PCT+LRRT**  **No. (%)** | **PCT+CCRT**  **No. (%)** | **P** |
| **Total** | 42 | 57 |  | 24 | 30 |  | 13 | 20 |  |
| **Bone involvement** |  |  |  |  |  |  |  |  |  |
| No | 13 (31.0) | 15 (26.3) | 0.613 | 13 (54.2) | 10 (33.3) | 0.124 | 2 (15.4) | 3 (15.0) | 1.000 |
| Yes | 29 (69.0) | 42 (73.7) |  | 11 (45.8) | 20 (66.7) |  | 11 (84.6) | 17 (85.0) |  |
| **Lung involvement** |  |  |  |  |  |  |  |  |  |
| No | 31 (73.8) | 47 (82.5) | 0.298 | 17 (70.8) | 25 (83.3) | 0.272 | 13 (100.0) | 10 (50.0) | 0.002 |
| Yes | 11 (26.2) | 10 (17.5) |  | 7 (29.2) | 5 (16.7) |  | 0 | 10 (50.0) |  |
| **Liver involvement** |  |  |  |  |  |  |  |  |  |
| No | 42 (100.0) | 57 (100.0) | NA | 12 (50.0) | 12 (40.0) | 0.462 | 8 (61.5) | 15 (75.0) | 0.461 |
| Yes | 0 | 0 |  | 12 (50.0) | 18 (60.0) |  | 5 (38.5) | 5 (25.0) |  |
| **Distant lymph node involvement** |  |  |  |  |  |  |  |  |  |
| No | 36 (85.7) | 48 (84.2) | 0.837 | 19 (79.2) | 28 (93.3) | 0.257 | 9 (69.2) | 13 (65.0) | 1.000 |
| Yes | 6 (14.3) | 9 (15.8) |  | 5 (20.8) | 2 (6.7) |  | 4 (30.8) | 7 (35.0) |  |
| **Number of involved organs** |  |  |  |  |  |  |  |  |  |
| Single | 37 (88.1) | 53 (93.0) | 0.630 | 16 (66.7) | 17 (56.7) | 0.454 | 6 (46.2) | 9 (45.0) | 1.000 |
| Multiple | 5 (11.9) | 4 (7.0) |  | 8 (33.3) | 13 (43.3) |  | 7 (53.8) | 11 (55.0) |  |
| **Number of involved lesions** |  |  |  |  |  |  |  |  |  |
| ≤ 4 | 42 (100.0) | 57 (100.0) | NA | 10 (41.7) | 13 (43.3) | 0.902 | 0 | 0 | NA |
| > 4 | 0 | 0 |  | 14 (58.3) | 17 (46.7) |  | 13 (100.0) | 20 (100.0) |  |
| **EBV-DNA status** |  |  |  |  |  |  |  |  |  |
| EBV-DNA ≤ 62000 | 29 (69.0) | 47 (82.5) | 0.118 | 23 (95.8) | 26 (86.7) | 0.367 | 0 | 0 | NA |
| EBV-DNA > 62000 | 13 (31.0) | 10 (17.5) |  | 1 (4.2) | 4 (13.3) |  | 13 (100.0) | 20 (100.0) |  |
| **Tumor response to PCT** |  |  |  |  |  |  |  |  |  |
| PR/CR | 31 (73.8) | 39 (68.4) | 0.560 | 20 (83.3) | 22 (73.3) | 0.583 | 6 (46.2) | 11 (55.0) | 0.728 |
| PD/SD | 11 (26.2) | 18 (31.6) |  | 4 (16.7) | 8 (26.7) |  | 7 (53.8) | 9 (45.0) |  |
| **Chemotherapy cycle** |  |  |  |  |  |  |  |  |  |
| < 4 | 2 (4.8) | 6 (10.5) | 0.505 | 1 (4.2) | 0 | 0.444 | 0 | 1 (5.0) | 1.000 |
| ≥ 4 | 40 (95.2) | 51 (89.5) |  | 23 (95.8) | 30 (100.0) |  | 13 (100.0) | 19 (95.0) |  |
| **Radiotherapy dose of loco-regional** |  |  |  |  |  |  |  |  |  |
| < 66Gy | 0 | 1 (1.8) | 1.000 | 0 | 0 | NA | 0 | 0 | NA |
| ≥ 66Gy | 42 (100.0) | 56 (98.2) |  | 24 (100.0) | 30 (100.0) |  | 13 (100.0) | 20 (100.0) |  |

*NPC* nasopharyngeal carcinoma, *PCT* palliative chemotherapy, *IMRT* intensity-modulated radiotherapy, *LRRT* locoregional intensity-modulated radiotherapy, *CCT* concurrent chemotherapy, CCRT concurrent chemoradiotherapy, *EBV* Epstein–Barr virus, *No.* Number, *NA* not applicable, *CR* complete response, *PR* partial response, *PD* disease progression, *SD* stable disease.
